# Potentiality of raloxifene loaded melittin functionalized lipidic nanovesicles against pancreatic cancer cells

**DOI:** 10.1080/10717544.2022.2072544

**Published:** 2022-06-16

**Authors:** Usama A. Fahmy, Shaimaa M. Badr-Eldin, Hibah M. Aldawsari, Nabil A. Alhakamy, Osama A. A. Ahmed, Mohamed F. Radwan, Basma G. Eid, Shaban R. M. Sayed, Gamal A. El Sherbiny, Walaa Abualsunun

**Affiliations:** a Department of Pharmaceutics, Faculty of Pharmacy, King Abdulaziz University, Jeddah, Saudi Arabia; b Department of Pharmaceutics and Industrial Pharmacy, Cairo University, Cairo, Egypt; c Center of Excellence for Drug Research and Pharmaceutical Industries, King Abdulaziz University, Jeddah, Saudi Arabia; d Mohamed Saeed Tamer Chair for Pharmaceutical Industries, King Abdulaziz University, Jeddah, Saudi Arabia; e Department of Chemistry, Faculty of Pharmacy, King Abdulaziz University, Jeddah, Saudi Arabia; f Department of Pharmacology, Faculty of Pharmacy, King Abdulaziz University, Jeddah, Saudi Arabia; g College of Science, Electron Microscope Unit, King Saud University, Riyadh, Saudi Arabia; h Department of Pharmacology, Faculty of Pharmacy, Cyprus International University, Nicosia, Cyprus

**Keywords:** Liposomes, ultrasonication, PANC1, apoptosis, membrane potential

## Abstract

Pancreatic cancer (PC) frequency and incidence have grown rapidly in recent years. One of the most serious problems with PC is the existence of asymptotic manifestations, which frequently delays early detection, and until the diagnosis is established, tumor cells progress to the metastatic stage. Another significant concern with PC is the scarcity of well-defined pharmacotherapeutic drugs. The aim of this study was to develop an efficient nanocarrier system to augment the efficacy of raloxifene (RLX) against PC cells. As a result, the current investigation was carried out in order to give an effective treatment method, in which an optimum RLX loaded phospholipid-based vesicles with melittin (PL-MEL) was chosen using experimental design software, with particle size, zeta potential and entrapment efficiency % as dependent variables. Furthermore, anticancer activity against PANC1 cells was assessed. The optimized nanovesicle parameters were 172.5 nm for the measured size, zeta potential of –0.69 mV, and entrapment efficiency of 76.91% that were in good agreement with the expected ones. RLX-raw, plain formula, and optimized RLX-PL-MEL showed IC_50_ concentrations of 26.07 ± 0.98, 9.166 ± 0.34, and 1.24 ± 0.05 µg/mL, respectively. Furthermore, cell cycle analysis revealed that the nanovesicle was most effective in the G2-M phase, whereas Bax, and Bcl-2 estimates revealed that optimized RLX formula had the highest apoptotic activity among treatments investigated. However, as compared to RLX alone or plain formula alone, the optimized formula demonstrated higher expression of TNFα and Bax while a significant reduction of Bcl-2 and NF-κB expression was observed. mitochondrial membrane potential (MMP) analysis confirmed the apoptosis as well as the anticancer effect of the optimized formula. Thus, the present study results showed an improvement in the anti-PC effects of the RLX with phospholipid conjugated melittin, making it a novel treatment approach against PC.

## 1. Introduction

In developing countries, pancreatic carcinoma is one of the most frequent types of cancer (Hani et al., 2021). It is the seventh in terms of cancer related deaths and ranked 13 in terms of number of new cases in 2020 worldwide (Li et al., 2019). Despite ongoing research, pancreatic cancer (PC) morbidity and mortality remain high. The disease is becoming more frequent in younger people (Barbier et al., 2011; Ilic & Ilic, 2016; Wang et al., 2019). This high statistic is related to the fact that the majority of PC patients have no visible symptoms until the disease advances to advanced pancreatic metastasis, when tumor cells are highly invasive (Zhao & Liu, 2020). Consequently, a better understanding of PC’s prevalence and progression is critical for early detection and treatment (Wang et al., 2019).

Cancer treatment by chemotherapeutic agents is accompanied by many disadvantages include nonselective toxicity toward the human cell, low accumulation inside the tumor and less responsiveness due to drug resistance (Hashemi et al., 2020). In the field of cancer therapy, nanoparticles were quickly developed and being launched in an attempt to address many limitations of traditional small-molecule chemotherapeutics (Shapira et al., 2011).

Raloxifene (RLX) hydrochloride is the first medication from the selective estrogen-receptor modulators family to be licensed by the US Food and Drug Administration as a preventative and therapeutic agent for osteoporosis in postmenopausal women and for the prevention of breast malignant tumors (O’Donnell et al., 2014; FDA Raloxifene Breast Cancer, 2022; National Cancer Institute, 2022). RLX has been shown to boost gene promoters controlled by the ER subtype, which protects against tumor development in response to estrogen, and hence has anti-tumor effect in breast cancer cells (Kim et al., 2015; Aldawsari et al., 2021). More recently, research have been directed to study the feasibility of using the medicine for other types of cancer therapy (Muchmore, 2000; Pritchard et al., 2016; Almutairi et al., 2019). Recent reports have indicated the efficacy of RLX against prostate cancer (Pritchard et al., 2016; Badejo et al., 2017; Asadirad et al., 2019; Pozios et al., 2021). Nonetheless, RLX has low water solubility and is subjected to severe pre-systemic metabolism, resulting in decreased bioavailability (Hochner-Celnikier, 1999; Mizuma, 2009; Kokawa et al., 2013).

Melittin (MEL) is a 26-amino acid short linear water-soluble cationic peptide with no disulfide bridge that is the main component (40–60% of the dry weight) and the predominant pain-inducing chemical in honeybee (*Apis mellifera*, European honey bee) venom (Lima et al., 2020). This peptide has shown promise in wound healing, oxidative stress, apoptosis, inflammation, antiviral, and multi-resistant bacterial infection (Moreno & Giralt, 2015; Mahmoudi et al., 2020; Memariani et al., 2020; Lima et al., 2021; Eid et al., 2022). Various reports have indicated promising anticancer activity of MEL (Gajski & Garaj-Vrhovac, 2013; Liu et al., 2016; Rady et al., 2017; Wang et al., 2022).

MEL also prevents cancer cells from invading and spreading. MEL inhibits the ras-related C3 botulinum toxin substrate 1 (Rac1) (Choi et al., 2014), which participates in the c-Jun N-terminal kinase (JNK) and JNK-dependent cell motility processes and causes metastasis, preventing hepatocellular carcinoma cell metastasis. MEL also specifically inhibits matrix metalloproteinase-9 (MMP-9) production, which is crucial for cancer cell motility (Jo et al., 2012; Vago et al., 2016), by downregulating activator protein-1 (AP-1) and NF-B expression (Pike et al., 1999; Moon et al., 2006).

Due to its wide range of biological and pharmacological effects, liposomes are nano particles stabilized by a phospholipid (PL) envelope creating a bilayer at the aqueous interface (Fenske & Cullis, 2008; Malam et al., 2009; Akbarzadeh et al., 2013; Ahmed et al., 2017; Alavi et al., 2017; Rai et al., 2020). This distinguishing feature allows for the encapsulation of larger concentrations of lipophilic drugs with prolonged release (Raju et al., 2013; Thoniyot et al., 2015; Al Asmari et al., 2016; Bardania et al., 2019). Lipophilic drugs are more soluble and bioavailable when they are encased in a PL lipid bilayer (Chen et al., 2009; Yokoyama, 2010; Elizondo et al., 2011). Furthermore, the site specificity of liposomes is regarded an additional benefit in cancer therapy due to their nano-size, which promotes drug targeting activity (Torchilin & Levchenko, 2003; Mandal et al., 2016; Rideau et al., 2018; Tajvar et al., 2019). As a result, the current study aims to develop and optimize RLX-MEL liposomes to offer controlled release and increased anti-cancer action of the drug. The modified liposomal formulation with reduced vesicle size and release efficiency, as well as increased zeta potential absolute value and drug entrapment, was tested for anti-tumor effectiveness in human PC cells *in vitro*. The aim of this study was to develop an efficient nanocarrier system to augment the efficacy of RLX against PC cells.

## 2. Materials and methods

Raloxifene (RLX) hydrochloride was from Qingdao Sigma Chemical Co., Ltd. (Qingdao, China). Cholesterol, MEL, fetal bovine serum (FBS), cell viability kit and high-glucose Dulbecco’s modified Eagle medium (DMEM) were purchased from Sigma-Aldrich (St. Louis, MO) were purchased from Sigma-Aldrich Inc. (St. Louis, MO). Phospholipid was Lipoid^®^90 H that was kindly gifted by Lipoid GmbH (Ludwigshafen, Germany). The human pancreatic cancer cell line (PANC1) isolated from a pancreatic carcinoma used in this study was obtained from the VACSERA (Giza, Egypt) cell culture unit that was originally acquired from American Type Culture Collection (Manassas, VA).

### 2.1. Experimental design for RLX-PL-MEL nanovesicles

RLX-PL-MEL nanovesicles were developed using response surface, specifically face-centered central composite design; Design-Expert software was utilized in this process (12.0; Stat-Ease Inc., Minneapolis, MN). The effects of two independent numerical factors, namely PL concentration (*X*_1_, mg/mL), and MEL concentration (*X*_2_, mg/mL) on particle size (*Y*_1_, nm), zeta potential (*Y*_2_, mV), and entrapment efficiency (*Y*_3_, %) as responses were examined. The levels of the examined factors are shown in Table 1.

**Table 1.** Independent variables’ levels and responses’ constraints used in the central composite design for the optimization of RLX-PL-MEL nanovesicles.

Independent variables	Levels		
	(–1)	(0)	(+1)
*X*_1_: PL concentration (mg/mL)	20	40	60
*X*_2_: MEL concentration (mg/mL)	5	10	15

Responses	Desirability constraint
*Y*_1_: particle size (nm)	Minimize
*Y*_2_: Zeta potential (mV)	Maximize
*Y*_3_: entrapment efficiency (%)	Maximize

RLX: raloxifene; PL: phospholipid; MEL: melittin.

The software generated 13 experimental trials as per the design. Table 2 shows the combined levels of the independent variable in each experimental run, as well as the measured responses. The model fit statistical analysis was performed to select the best-fitting sequential model for each response. The highest adjusted and predicted *R*^2^ and the least predicted residual sum of squares (PRESS) were used to decide the optimal model. To show the influence of the variables and their interactions, 2D contour and 3D surface plots were created.

**Table 2.** Combination of independent variables in RLX-PL-MEL nanovesicles experimental runs and their corresponding measured responses.

Run	PL concentration (*X*_1_, mg/mL)	MEL concentration (*X*_2_, mg/mL)	Particle size (*Y*_1_, nm)	Zeta potential (*Y*_2_, mV)	Entrapment efficiency (*Y*_3_, %)
1	40	10	243	–10.6	78.7
2	60	5	275	–19.3	84.6
3	40	10	241	–9.9	77.7
4	20	10	175	–1.4	73.7
5	20	15	188	3.1	74.7
6	40	10	239	–10.9	76.8
7	20	5	156	–4.6	74.6
8	40	10	242	–10.3	77.5
9	60	15	319	–14.1	88.8
10	40	5	216	–12.8	74.8
11	60	10	298	–16.5	87.4
12	40	10	244	–9.7	77.4
13	40	15	265	–8.1	76.9

RLX: raloxifene; PL: phospholipid; MEL: melittin.

### 2.2. Optimization of RLX-PL-MEL nanovesicles

For the optimization of RLX-PL-MEL nanovesicles, a numerical technique incorporating the desirability function was used. Based on the goals of decreasing particle size, maximizing zeta potential and entrapment efficiency, the optimal amounts of the researched independent variables were predicted. The optimized formulation was then prepared for investigation of *in vitro* anticancer activity.

### 2.3. RLX-PL-MEL preparation

Nanovesicles were prepared using the thin-film hydration method (Sinico et al., 2005). Phospholipid (as specified in the design), cholesterol (150 mg), and RLX (50 mg) were dissolved in 25 mL of a chloroform–methanol mixture (4:1) at a constant molar ratio. To eliminate residues of solvent and produce a film, the mixtures were evaporated in a rotary evaporator (IKA RV10 Digital Rotary Evaporator, IKA Pvt. Ltd., Bangalore, India) for 15 minutes at a speed of 70 rpm and a temperature of 46 °C. The film was hydrated for one hour at room temperature with phosphate buffer (10 mL, pH 7.4), which was above the lipid transition temperature. A probe sonicator was used to homogenize the vesicle dispersion, which was subsequently passed through a 0.45 mm filter (Minisart CA 26 mm). MEL (according to the design) was included in the dispersion, stirred for 10 min at 400 rpm using a magnetic stirrer and then stored until use.

### 2.4. Particle size, zeta potential entrapment efficiency measurements

Using a Nano-ZSP particle size analyzer, the particle size of RLX-PL-MEL (*z*-average) and zeta potential values were determined (Malvern Instrument, Worcestershire, UK). Prior to measurement, samples were diluted sufficiently with the formulation’s aqueous phase to achieve an optimal count of 50–200 kilo-counts per second. An average of five measurements was used to calculate the mean particle size. For zeta potential measurement, diluted sample was loaded into disposable (DTS1070) cuvette and measured for zeta potential values.

Entrapment efficiency was measured as previously reported (Aldawsari et al., 2021). Briefly, indirect method was applied, 2 mL of RLX-PL-MEL was ultracentrifuged at 100,000 rpm for 1 h at 4 °C and the residue was washed twice with phosphate buffer and re-centrifuged again for 1 h. RLX concentration was determined in the combined supernatant by HPLC method as previously reported (Trontelj et al., 2005). RLX loading content (%) for the optimized formula was calculated as previously reported (Xu et al., 2013). All determinations were performed in triplicate.

### 2.5. Characterization of optimized RLX-PL-MEL

The optimized RLX-PLA-MEL was explored using transmission electron microscope (TEM) (JEOL GEM-1010, JEOL Ltd., Akishima, Japan) at 80 kV at Al-Azhar University’s Regional Center for Mycology and Biotechnology (RCMB). The studied materials were dispersed in water, and one drop of each was distributed on a carbon-coated grid before drying at room temperature. In addition, 1% phosphotungstic acid was utilized to stain the sample negatively. The material was then dried for 15 minutes at room temperature before being visualized.

Three freeze–thaw cycles (between –20 °C and +25 °C) were undertaken to test the stability of the optimized RLX-PLA-MEL formula. After that, the particle size was measured and compared to freshly created formulae.

Fourier-transform infrared (FTIR) spectra analysis of RLX, MEL, cholesterol, PL, and RLX-PL-MEL was utilized to investigate the possible interaction between optimized formula components measured in the range of 4000–400 cm^–1^ using FTIR Tensor37 (Bruker, Billerica, MA).

To compare the release profile of the optimized RLX-PL-MEL formula to that of the RLX-raw drug suspension, 2 mg of RLX-raw suspension and equivalent concentration of the optimized formula were kept in separate dialysis bags (molecular weight cutoff 12 kDa). Then, with moderate agitation, the dialysis bags were submerged in 500 mL phosphate-buffered saline (PBS) (pH 7.4). At a predetermined time interval 0.5, 1, 2, 4, 8, 10, 12, 24 h, 1 mL of sample was taken from the dissolution medium and immediately replaced with the same volume of fresh buffer. Following that, collected samples were measured by HPLC (Trontelj et al., 2005) to determine the cumulative RLX released.

### 2.6. Cytotoxicity study of optimized RLX-PL-MEL via MTT assay

The potential cytotoxic effect of optimized RLX-PL-MEL was studied using the PANC1 cell line and MTT assay. Cells were grown in DMEM, 10% FBS, 100 units/mL penicillin, 100 μg/mL streptomycin in 5% CO_2_ and at 37 °C. The screened PANC1 cells were transferred to the 96-well plates (5 × 10^3^/cells/well) and overnight incubated. Once cells were stabilized, the plates were exposed to RLX-raw, plain formula (PL-MEL), and RLX-PL-MEL and left for incubation for 48 h. After 48 h, the treated cells were exposed to 10 µL of MTT solution (5.0 mg/mL) and again incubated for 4 h at 37 °C. After 4 h, the supernatants were collected and treated with 100 mL DMSO. The obtained samples were evaluated at 570 nm using microplate readers (Alhakamy et al., 2021).

### 2.7. Cell cycle analysis of optimized RLX-PL-MEL

The effects of different sample formulations such as RLX, plain formula, and RLX-PL-MEL on cell cycle analysis were determined by using flow cytometric analysis. For this purpose, the PANC1 cells were treated with different samples and left for incubation for 24 h. After 24 h, cells were centrifuged and treated with 70% cold ethanol, washed, and again centrifuged. Propidium iodide and RNAse were mixed with the obtained separated cells before proceeding for flow cytometric analysis (Luna-Vital et al., 2016; Alhakamy et al., 2020; Awan et al., 2020).

### 2.8. Real-time polymerase chain reaction (RT-PCR) for estimation of Bcl-2, Bax, TNF-α, and NF-κB

The expression of Bcl-2, Bax, and TNF-α was determined by using RT-PCR. The PANC1 cells were treated with RLX-raw, Plain formula, and RLX-PL-MEL and incubated for the specified period. The cell fraction was used for the extraction of RNA and proceeded for the synthesis of cDNA. Primer for the Bcl-2, Bax, TNF-α, and NF-κB was designed by using Gene Runner software (Table 3). The prepared samples were estimated for the expression in triplicate, and the samples were normalized with the help of β actin (Alhakamy et al., 2020, 2021).

**Table 3.** Sequences of nucleotides for the primers used in the analysis of mRNA expression by RT-PCR.

Gene	Primer sequence
β-actin	F 5′-GCACCACACCTTCTACAATG-3′
	R 5′-TGCTTGCTGATCCACATCTG-3′
Bcl-2	F 5′-ATGTGTGTGGAGACCGTCAA-3′,
	R 5′-GCCGTACAGTTCCACAAAGG-3′.
Bax	R: 5′-TCACCAACTGGGACGATATG-3′
	F: 5′- TCCGTCGCCGGTCCACACCC-3′
NF-κB	F 5′-GCAGCACTACTTCTTGACCACC-3′,
	R 5′-TCTGCTCCTGAGCATTGACGTC-3′.
TNF-α	F 5′-CTCTTCTGCCTGCTGCACTTTG-3′,
	R 5′-ATGGGCTACAGGCTTGTCACTC-3′.

### 2.9. Determination of mitochondrial membrane potential (MMP)

MMP was assessed using the ABCAM test kit (Cambridge, UK). Cells were incubated for 24 hours on a 96-well plate with PANC1 cell density was 5 × 10^3^. Separately, the RLX-raw, plain formula, and the optimized formula were introduced to the wells. The resulting cell combination was put in the dark, the probe solution (tetramethylrhodamine, methyl ester) was replenished, and MMP was measured using a BD Bioscience FACS Caliber flow cytometer (Xue et al., 2014; Hussain, 2019).

### 2.10. Statistical analysis

The value was presented as the mean ± standard deviation (SD). For statistical analysis, one-way ANOVA was employed, followed by Tukey’s multiple comparison test, with a *p* value of .05 considered significant.

## 3. Results

### 3.1. Experimental design

Table 4 summarizes the results of the fit statistical analysis for the measured particle size, zeta potential, and entrapment efficiency %. All the responses fitted the quadratic model according to the highest computed *R*^2^ and lowest PRESS among the different polynomial sequential models. For the three responses, the adjusted and the predicted *R*^2^ showed good coincidence, where the difference between them was less than 0.2. In addition, the adequate precision values were markedly higher than the desirable value of 4 (85.77, 54.85, and 24.91 for particle size, zeta potential, and entrapment efficiency %, respectively) indicating a good signal to noise ratio. Thus, the quadratic model could be regarded as adequate model for exploration of design space.

**Table 4.** Fit summary statistics for RLX-PL-MEL nanovesicles’ responses according to the quadratic model.

Response	Sequential *p* value	Lack of fit *p* value	*R* ^2^	Adjusted *R*^2^	Predicted *R*^2^	PRESS
Particle size	.0144	.1156	0.9978	0.9963	0.9828	448.14
Zeta potential	.0064	.2730	0.9946	0.9907	0.9652	14.99
Entrapment efficiency	.0005	.2124	0.9821	0.9693	0.9021	28.96

RLX: raloxifene; PL: phospholipid; MEL: melittin; PRESS: predicted residual error sum of squares; 2FI: two-factor interaction.

**Figure 1.**
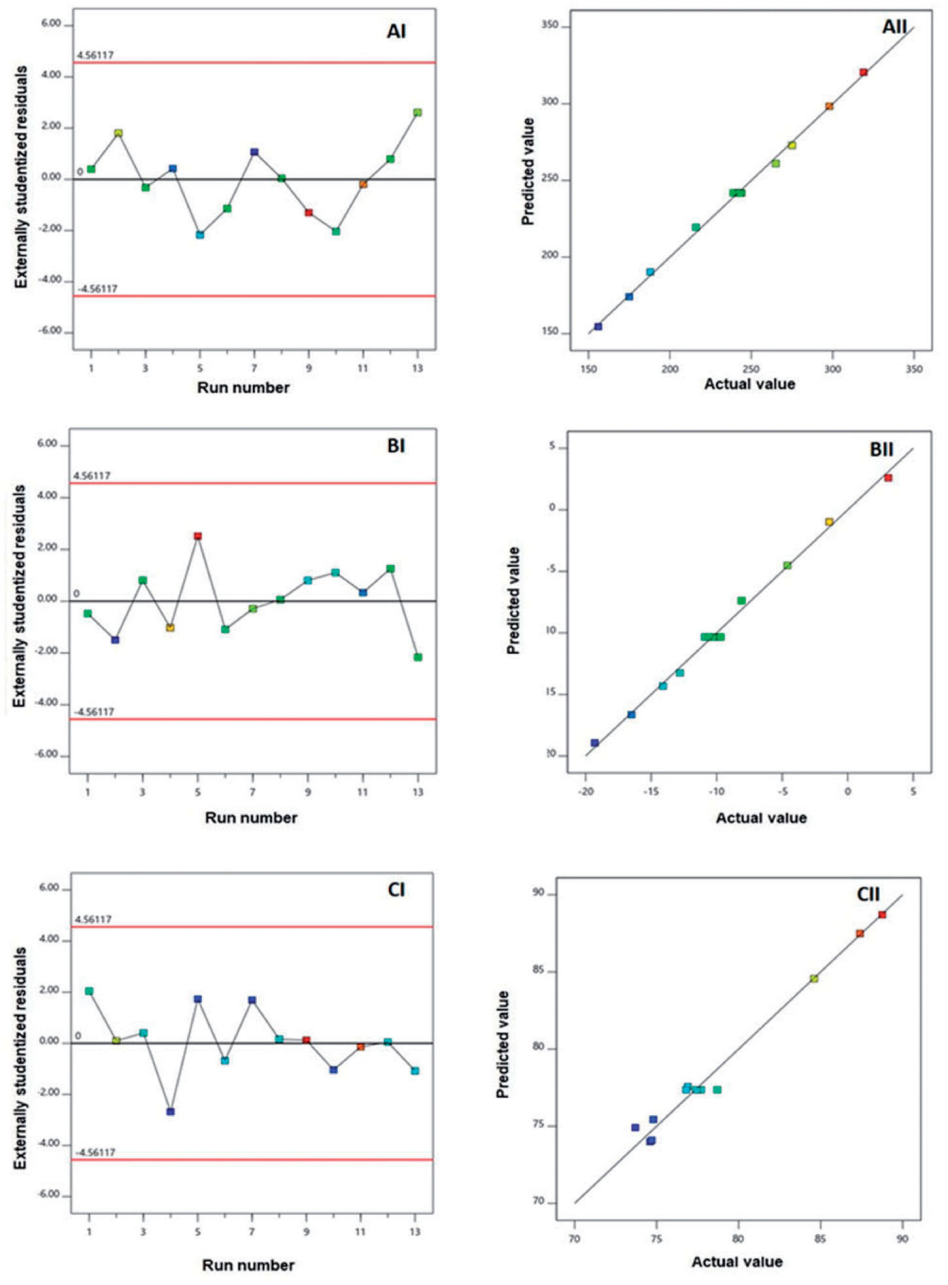
Diagnostic plots for (A) vesicle size, (B) zeta potential, and (C) entrapment efficiency of RLX-PL-MEL nanovesicles. (I) Externally studentized residuals vs. run number plots and (II) predicted vs. actual responses.

Diagnostic plots, as shown in Figure 1, were created to determine the goodness of fit of the chosen models. Figure 1(AI, BI, and CI) provides externally studentized residuals vs. run plots with randomly spread dots, suggesting that no lurking variable could impact the measured responses. Figure 1(BI, BII, and BIII) also shows high linearity, showing a good correlation between expected and actual values for all responses and, as a result, the acceptability of the quadratic model (Fahmy et al., 2020; Badr-Eldin et al., 2021).

### 3.2. Influence of investigated variables on PS (*Y*_1_)

To target malignant tumor penetration, research aimed at changing drug delivery system features, specifically the particle size of multi-particulate systems, is ongoing and substantial (Han et al., 2018). Nanoscale systems have lately emerged as a promising technique in the field of cancer therapy. Particles smaller than 400 nm have been found to accumulate preferentially within solid malignant tissues (Sharma et al., 2014; Yingchoncharoen et al., 2016). The prepared RLX-PL-MEL nanovesicles showed appropriate average size ranging from 156 to 319 nm. However, inadequate penetration into malignant tissue due to pathological circumstances established by malignancy may counteract the preferential absorption of nano-sized systems and their related therapeutic efficacy (Zhang et al., 2019). Reducing vesicle size to enhance the region suitable for permeation could help increase tumor penetration (Badr-Eldin et al., 2021). As a result, the proposed RLX-PL-MEL nanovesicles’ size was optimized to ensure efficient tumor penetration.

For statistical analysis of the measured PS, analysis of variance was used. The estimated *F*-value of 639.87 (*p*<.0001) confirmed the quadratic model’s significance. The lack of fit *F*-value of 3.78 (*p*=.1156) demonstrated non-significant lack of fit when compared to pure error, indicating that fitting to the proposed model. Equation (1) representing the quadratic model for PS in terms of coded factor was generated by the Design-Expert software (Stat-Ease Inc., Minneapolis, MN).

(1) $$Y_{1}\;\left(\text{vesicle}\text{size}\right)=241.90+62.17X_{1}+20.83X_{2}-5.64X_{1}X_{2}-5.74X_{1}{}^{2}-1.64X_{2}{}^{2}$$

**Figure 2.**
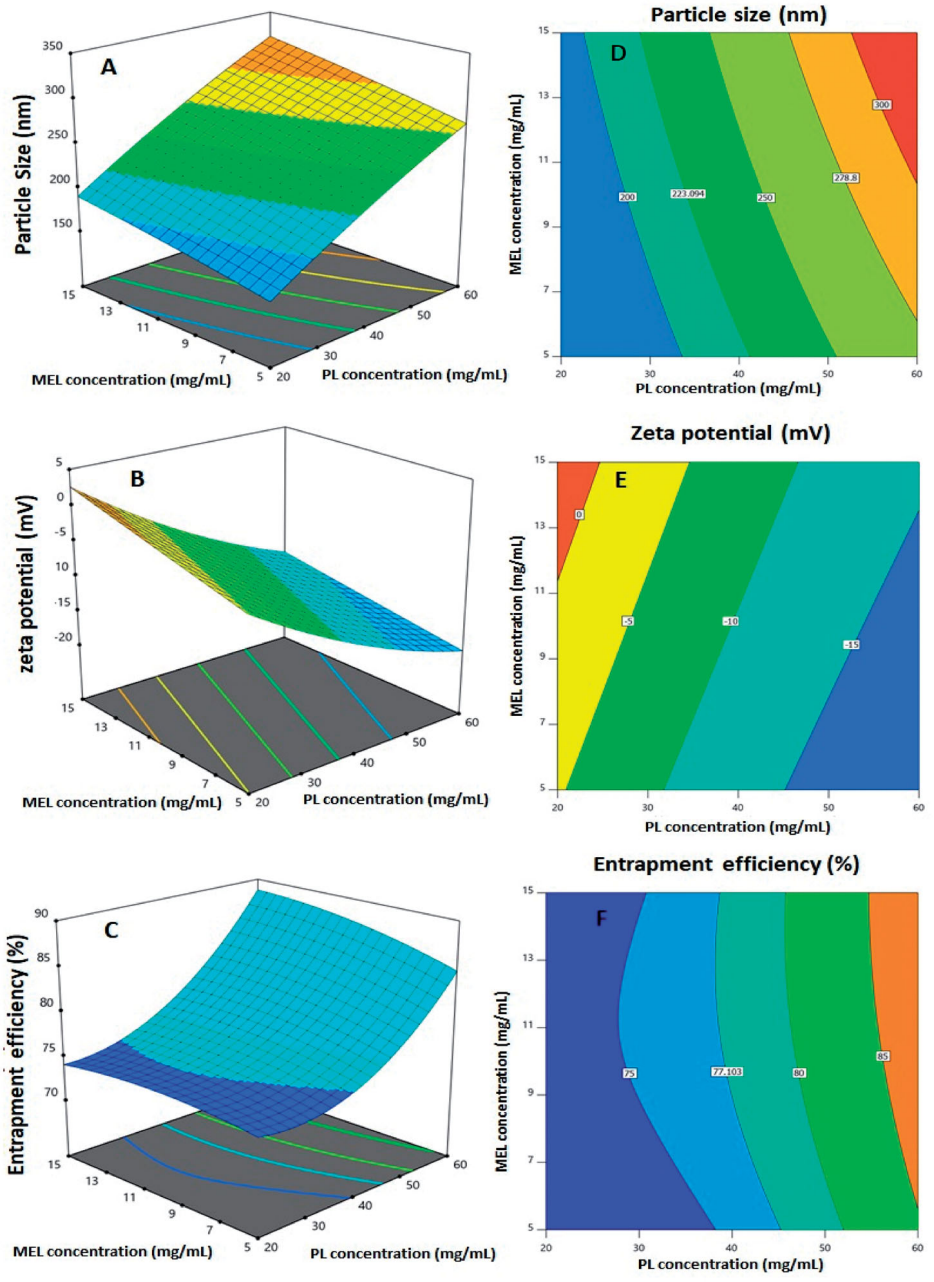
Response 3D-plots (A–C) and contour 2D-plots (D–F) for the influence of PL concentration (*X*_1_) and MEL concentration (*X*_2_) on the responses of RLX-PL-MEL nanovesicles. RLX: raloxifene; PL: phospholipid; MEL: melittin.

**Figure 3.**
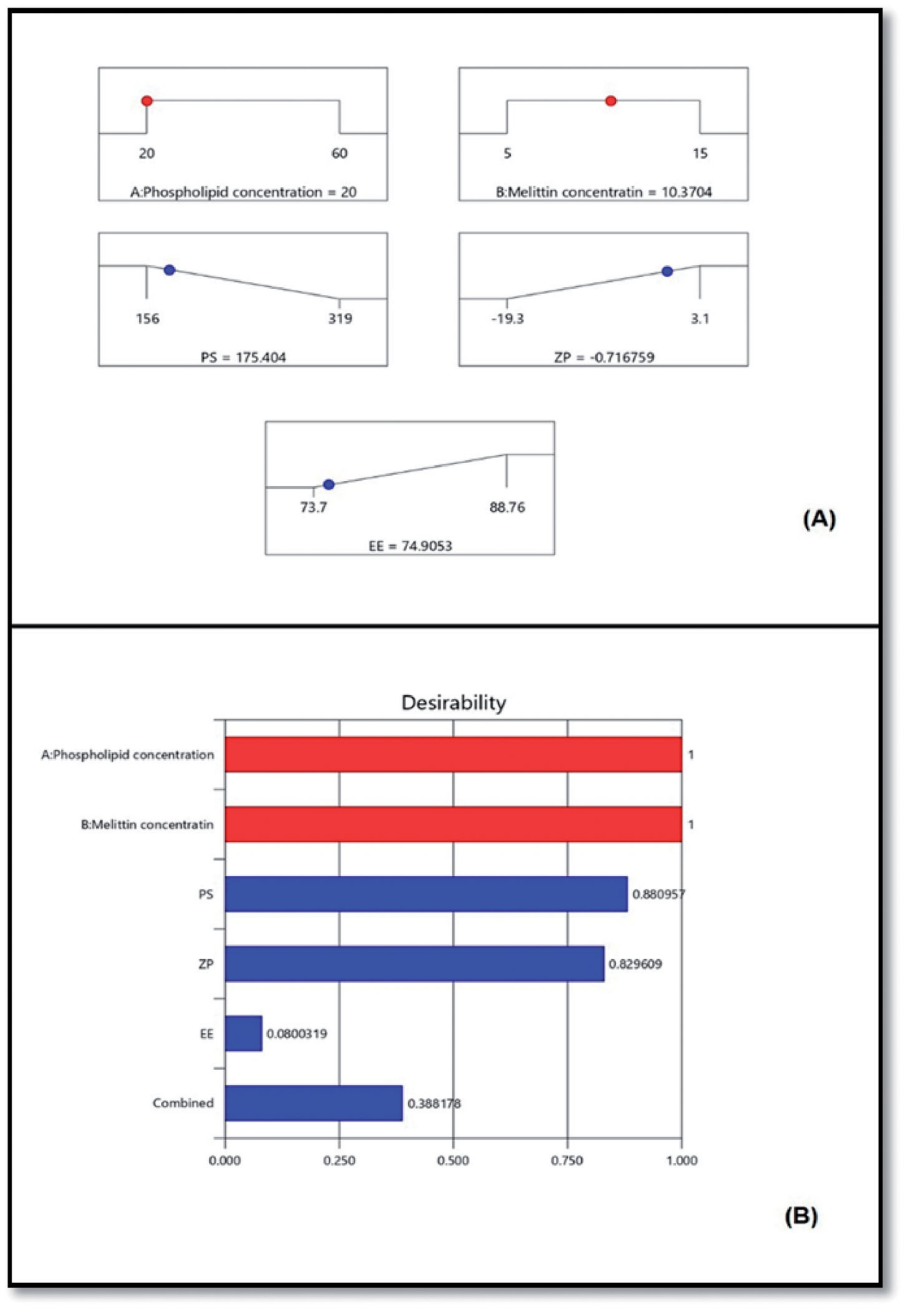
(A) Ramp graphs representing the optimized levels of the independent variables and the predicted responses for the optimized RLX-PL-MEL nanovesicles. (B) Desirability values for the predicted responses and overall desirability of the optimized RLX-PL-MEL nanovesicles. RLX: raloxifene; PL: phospholipid; MEL: melittin.

Both linear terms, *X*_1_ and *X*_2_, corresponding to the investigated variables, exhibited a significant effect on particle size (*p*< .0001). In addition, the quadratic term *X*_1_^2^ was also found to be significant (*p*= .0133). Figures 2 and 3 demonstrate the 2D-contour and the 3D-response plots representing the effects and interactions between the studied variables on both responses. The illustrations show that the PS significantly increases with increasing both PL and MEL concentrations. This finding is supported by the positive sign of *X*_1_ and *X*_2_ coefficients in the developed equation. The higher coefficient of the term *X*_1_ compared to that of *X*_2_ indicates that the impact of the PL concentration was more pronounced compared to MEL concentration. This result was in good coincidence with previous studies that proved the increase of vesicles size at higher PL levels (Awan et al., 2020).

### 3.3. Influence of investigated variables on zeta potential (*Y*_2_)

The zeta potential is considered as a surface charge indicator. Cationic systems have been shown to have much stronger permeability into malignant cells and better build up inside the tumor tissue and vasculature (Krasnici et al., 2003; Wang et al., 2016; Saadat et al., 2019). As a result, MEL was used to reduce the intensity of the negative charge on the vesicles. The proposed formulations exhibited ZP ranging from –19.3 to 3.1 mV.

Based on the analysis of variance results, the significance of the quadratic model for ZP is confirmed by the *F*-value of 256.91 (*p*= .0064). Non-significant lack of fit relative to pure error was proved by the lack of fit *F*-value of 1.89 (*p*= .2730); therefore, fitting to the suggested model is ensured. Equation (2) demonstrates the quadratic model in terms of coded factor was generated by the software.

(2) $$Y_{2}\;\left(\text{zeta}\text{potential}\right)=–10.33–7.83X_{1}+2.93X_{2}–0.625X_{1}X_{2}+1.52X_{1}{}^{2}+0.021X_{2}{}^{2}$$

Both linear terms, *X*_1_ and *X*_2_, corresponding to the investigated variables, exhibited a significant effect on zeta potential (*p*< .0001). Moreover, the quadratic term *X*_1_^2^ was also found to be significant (*p*= .0033). Figure 1(B,E) demonstrates the 3D-response and the 2D-contour plot representing the effects and interactions between the studied variables on ZP. The illustrations shows that the ZP significantly decreases with increasing PL concentration, while increases at higher MEL concentrations. This finding is supported by the positive sign of *X*_1_ and the negative sign of *X*_2_ coefficients in the developed equation. This could be explained on the basis of the anionic nature of the PLs and the positively charged groups present in MEL, and consequently its role in reducing the negative charge on the surface of nanovesicles (Ortiz et al., 2015; Liu et al., 2018).

### 3.4. Influence of investigated variables on EE% (*Y*_3_)

The prepared nanovesicles showed satisfying drug entrapment exceeding 70% for all formulations. The significance of the quadratic model for EE% is corroborated by the *F*-value of 66.68 (*p*=.0005) based on the analysis of variance data. Based on the non-significant lack of fit (*p*=.2124), fitting to the suggested model is assured. The following is the equation created by the Design Expert software (Stat-Ease Inc., Minneapolis, MN) to demonstrate the quadratic model for EE% in terms of coded factor:

$$Y_{3}\;\left(\text{EE}\%\right)=77.36+6.29X_{1}+1.06X_{2}+1.02X_{1}X_{2}+3.84X_{1}{}^{2}–0.86X_{2}{}^{2}$$

Both linear terms, *X*_1_ and *X*_2_, which corresponded to the factors studied, had a substantial effect on EE% (*p*<.0001 and *p*=.0202 for *X*_1_ and *X*_2_, respectively). Furthermore, the quadratic term *X*_1_^2^ (*p*=.0002) was found to be significant. The 3D-response and 2D-contour plots showing the effects and interactions between the examined factors on EE% are shown in Figure 1(C,F). The EE% increases proportionally with both PL and Mel concentrations, as shown in the figures. The positive signs of both *X*_1_ and *X*_2_ coefficients in the generated equation support this observation. The influence of PL concentration is more pronounced owing to the higher coefficient of *X*_1_. The observed higher entrapment at higher PL concentrations could be related to the increased size of the vesicles that could allow for more space for drug entrapment within the PL bilayer.

### 3.5. Optimization of RLX-PL-MEL nanovesicles

Using the numerical technique and the desirability approach, the levels of the optimized variables that might give the reduced size and maximized zeta potential when combined were anticipated. The ramp graphs in Figure 3(A) depicted the optimal values as well as the projected responses. Figure 3(B) shows the desirability of each response as well as the overall desirability values. With relative percentage errors of 1.65, 4.16, and 2.68%, respectively, the measured size of 172.5 nm, zeta potential of –0.69 mV, and entrapment efficiency of 76.91% that were in good agreement with the expected ones. The optimization process’s reliability is demonstrated by the low relative percentage error for all responses. In addition, the loading capacity (%) of the optimized RLX-PL-MEL nanovesicles showed 9.89%.

### 3.6. Characterization of optimized RLX-PL-MEL

**Figure 4.**
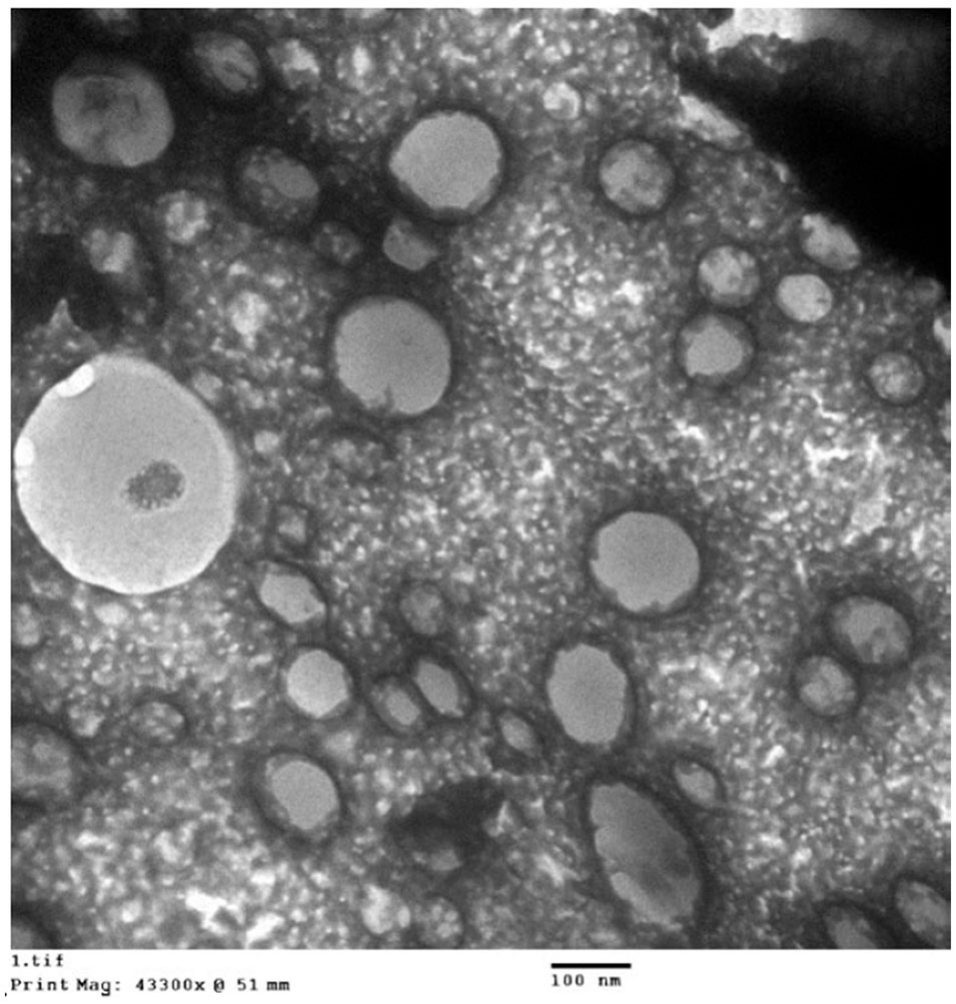
TEM image of optimized RLX-PL-MEL. RLX: raloxifene; PL: phospholipid; MEL: melittin.

The optimized RLX-PL-MEL was investigated utilizing TEM as shown in Figure 4. The figure revealed spherical nanovesicles with an average size (99.2 nm) relatively smaller than the size measured by the dynamic light scattering technique (172.5 nm). This could be attributed to the drying process in case of TEM sample during its preparation. In addition, the optimized formula showed no significant change (*p*<.05) regarding vesicle size after running three freeze–thaw cycles (between –20 °C and +25 °C) that can indicate stability of the optimized RLX-PL-MEL formula.

**Figure 5.**
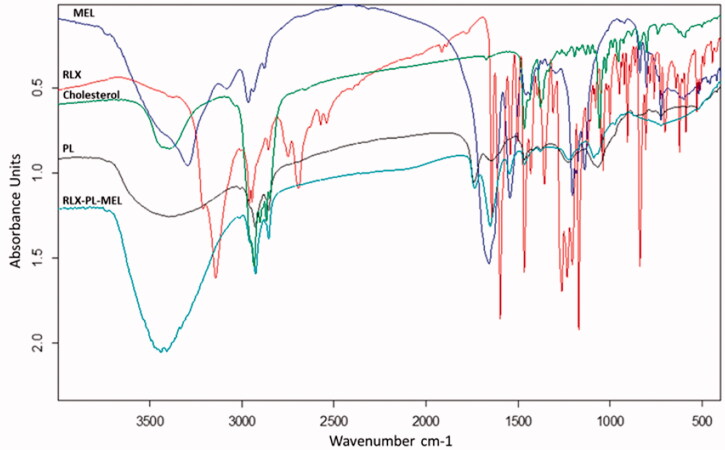
FTIR spectra of MEL, RLX, cholesterol, PL, and the optimized RLX-PL-MEL formula.

FTIR spectra of RLX, MEL, PL, cholesterol, and the optimized RLX-PL-MEL formula (Figure 5). RLX showed a peak around 3100 cm^–1^ related to C–H aromatic, sharp peak at 1700 cm^–1^ related to ketonic C=O, a C–O bond at 1100–1200 cm^–1^ and C–H aliphatic peak at around 2950 cm^–1^. MEL spectrum revealed a broad band at 3300–3400 cm^–1^ of nitrogenous functional groups. MEL spectra at 1600–1700 cm^–1^ showed a broad band related to the amide group of peptide backbone. In addition, at 1500–1600 cm^–1^ and 1100–1250 cm^–1^ peaks that were related to N–H bending vibration of NH_2_ and the C–O stretch vibrations from the C-terminal amino acid, respectively. Cholesterol showed a broad band at 3300–3400 cm^–1^ of O–H group, a very broad sharp peaks at 2800–2900 cm^–1^ for C–H aliphatic. PL revealed a very broad peak at 3300–3500 cm^–1^ of O–H group and C–H aliphatic before 3000 cm^–1^. PL also showed a peak at 1735 cm^–1^ for carbonylic C=O ester and C–O at 1100 cm^–1^.

**Figure 6.**
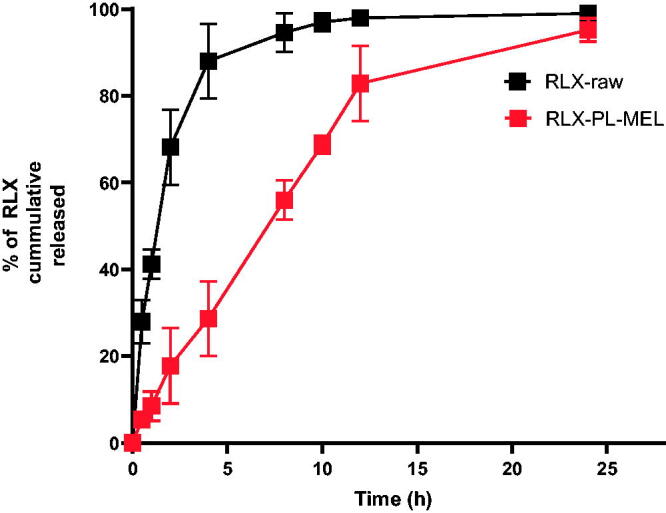
The *in vitro* release pattern of RLX from optimized RLX-PL-MEL and RLX-Raw.

The optimized RLX-PL-MEL showed broadening of peaks around 3500 cm^–1^ that indicates more interactions and involvement of hydroxyl and amino groups in more hydrogen bonding of all formula components. In addition, the augmentation of the C–H aliphatic peak around 2800 cm^–1^ that is related to the major aliphatic nature of formula components. The reduction of peak intensity around 1700 cm^–1^ that is related to the region of C=O group that may be attributed to dilution of component concentrations in the formula and participation as hydrogen bond acceptor for solvolysis of polar components of the optimized formula (Figure 5). The results of an *in vitro* release study showed that RLX was released faster in case of RLX-raw suspension, while RLX was delayed on release from the optimized formula. Figure 6 shows that the optimized formula released about 95% of its RLX over 24 h. However, more than 90% of the RLX-raw was released in 8 h.

### 3.7. Cell viability assay

**Figure 7.**
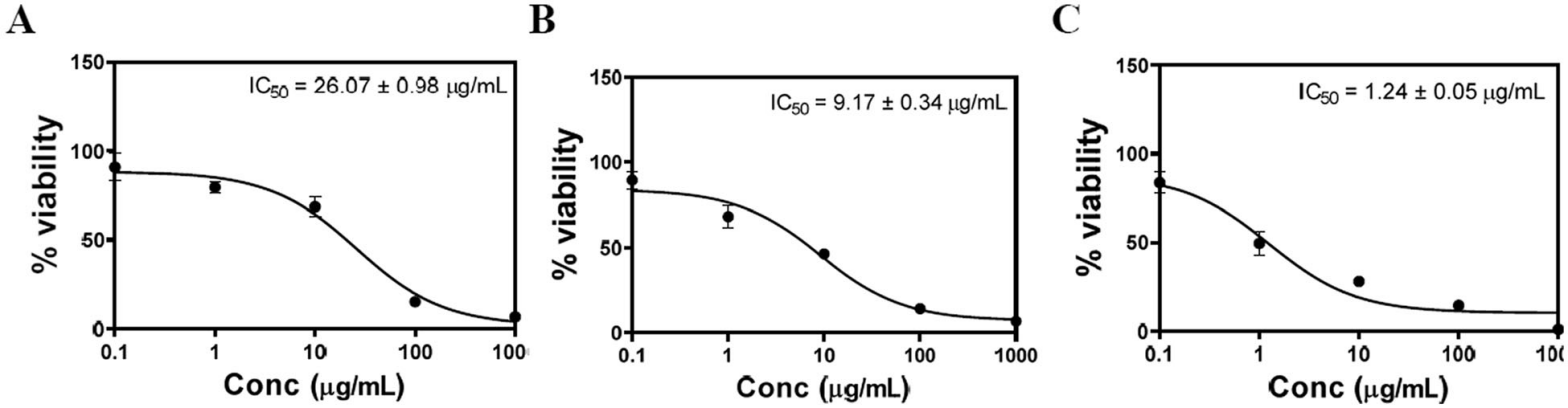
Comparison of PANC1 cell viability results using MTT assay. The cells were treated with PL-MEL (A), RLX (B), or RLX-PL-MEL (C) for 48 h.

MTT colorimetric assay was applied to evaluate the effect of PL-MEL, RLX, or RLX-PL-MEL treatment on the viability of PANC1 cells. After 48 h, plain PL-MEL formula did not show significant alteration in PANC1 cell growth. However, both RLX and RLX-PL-MEL significantly decreased PANC1 cell viability compared to PL-MEL plain formula with a concentration of 9.166 ± 0.34 µg/mL for RLX and 1.24 ± 0.05 µg/mL for RLX-PL-MEL (Figure 7). At this low concentration, RLX-PL-ME demonstrated a significant cytotoxic effect on PANC1 cells compared to PL-MEL and RLX (*p*<.0001) (Figure 7). This result confirms RLX-PL-ME antiproliferative effect on PANC1 cells.

### 3.8. Cell cycle analysis

**Figure 8.**
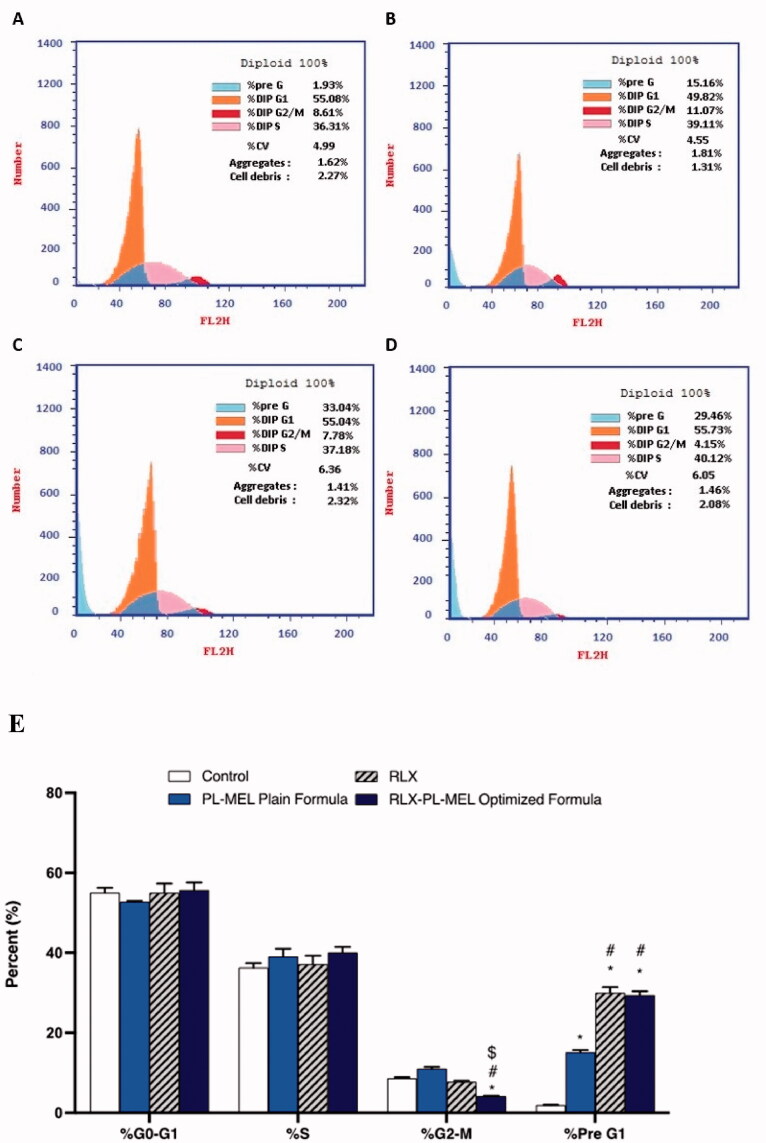
Cell cycle analysis of PANC1 cells using flow cytometry assay. PANC1 cells were treated with RLX-PL-MEL or RLX for 48 h. (A) control, (B) PL-MEL plain formula, (C) RLX, and (D) RLX-PL-MEL optimized formula. (E) Graphical presentation of each phase. Data are presented as percentage of mean ± SD of three independent experiments, *^,#,$^*p*<.0001. *Significant from control, ^#^significant from PL-MEL plain formula, and ^$^significant from RLX treated.

Cell cycles were analyzed in PANC1 cells treated with control, RLX, PL-MEL, or RLX-PL-MEL to confirm the antiproliferative efficacy of RLX-PL-MEL against PANC1 cells. The proliferative profile for control PANC1 cells was about 55% at G0/G1 phase, 36% at S phase, 8% at G2/M phase, and 4% at pre-G1-phase (Figure 8(A,E)). Following PL-MEL treatment, the percentage of cells in the G0/G1 phase slightly decreased 49.82±.17% compared to control 55.08 ± 1.2%. While treatment with RLX or RLX-PL-MEL did not show significant changes in the %G0/G1 phase.

However, an induction in the apoptotic pre G1 peak was detected following RLX (33.04 ± 1.40%), PL-MEL (15.16 ± 0.53%), and RLX-PL-MEL using 1.24 ± 0.05 µg/mL (29.46 ± 0.92%) respectively compared to control (1.93 ± 0.06%). This induction of apoptotic cells represents cell death before the beginning of the cell cycle.

Incubating PANC1 cells with RLX, PL-MEL, or RLX-PL-MEL did not cause a significant DNA accumulation in the S phase (37.18 ± 2.10%), (39.11 ± 1.9%), and (40.12 ± 1.4%), respectively. However, the percentage of cells in the G2/M phase decreased (4.15 ± 0.11%) following RLX-PL-MEL treatment when compared to RLX and PL-MEL, indicating rapid arresting activity (*p*>.0001). Thus, PANC1 treated with RLX-PL-MEL cell cycle analysis reveals slight incline in the G0/G1 phase, marked decrease in the G2/M phase and marked increase in the pre-G1 phase, all suggest a significant arrest of S phase and marked increases in the apoptotic effect compared to the treatment alone (Figure 8).

### 3.9. Bax and Bcl2 expression

**Figure 9.**
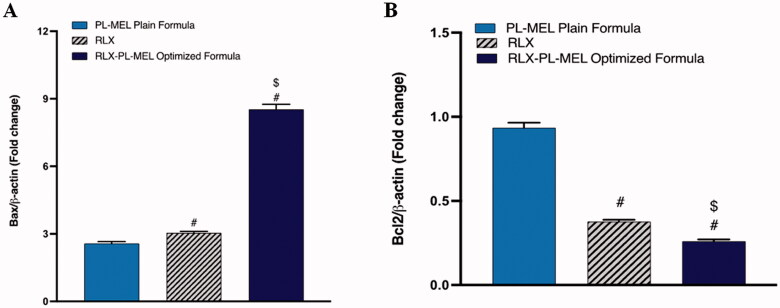
Effect of PL-MEL, RLX, or RLX-PL-MEL treatment on (A) Bax and (B) Bcl-2 proteins. Data are expressed as the fold change of mean ± SD of three independent experiments where ^#,$^*p*<.0001. ^#^Significant from PL-MEL plain formula, and ^$^significant from RLX treated. Samples were normalized to β actin.

To evaluate the therapeutic potential of the optimized formula RLX-PL-MEL, the expression of pro-apoptotic protein Bax was examined. Bax protein modulate the release of cytochrome *c*, alter MMP, and instigates rapid cell death. As shown in Figure 9(A), Bax expression was significantly increased in PAC1 treated with the optimized formula RLX-PL-MEL compared to RLX, and PL-MEL. Apoptosis was also confirmed by evaluating the expression of anti-apoptotic protein Bcl-2 (Figure 9(B)). This Bcl-2 protein hinders the release of cytochrome *c* from the mitochondria; therefore, the decrease in Bcl-2 expression under RLX-PL-MEL treatment indicates significant apoptotic effect compared to RLX, and PL-MEL.

### 3.10. TNF-α

**Figure 10.**
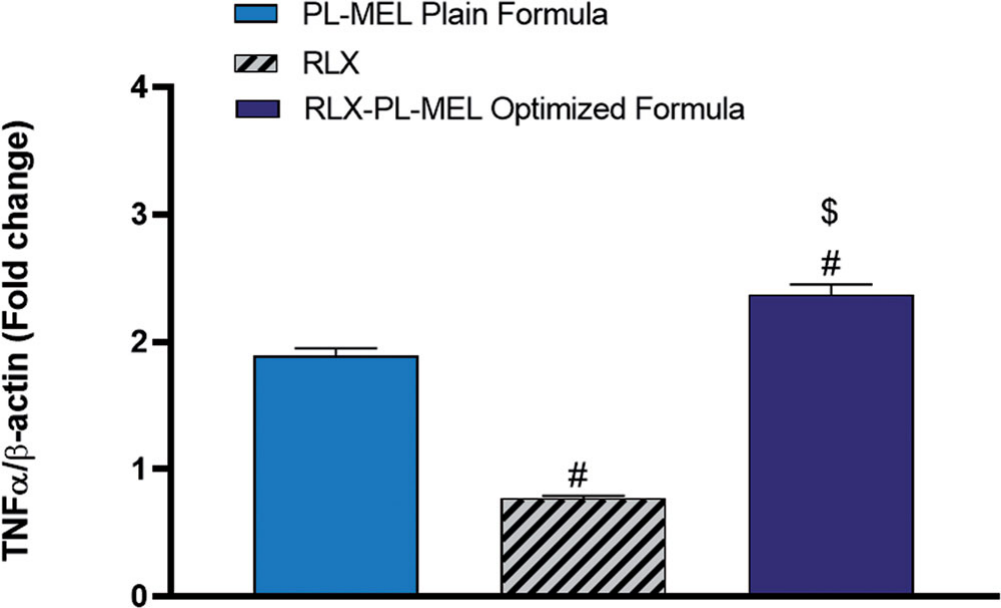
Effect of PL-MEL, RLX, or RLX-PL-MEL treatment on TNF-α activation. Data are expressed as the fold change of mean ± SD of three independent experiments where ^#,$^*p*<.0001. ^#^ Significant from PL-MEL plain formula, and ^$^significant from RLX treated. Samples were normalized to β actin.

Tumor necrosis factor α (TNF-α) is a double agent cytokine that plays a critical role in either cell survival or promoting cell death. However, in cancer cells, TNF-α receptor-1 (TNF-R1), which is expressed in virous tumorous cells including pancreatic cells, was shown to be responsible for transmitting the death signals from the cell surface to the intracellular signaling pathway following the activation of TNF-α (Elmore, 2007). TNF-α induced signaling pathways has been extensively studied in caner biology (Wang & Lin, 2008). Upon activation with TNF-α, TNF-R1 recruits the adaptor protein TNF-R1 associated death domain protein and its downstream caspase-8 causing apoptosis (Josephs et al., 2018). Undeniably, PANC1 cells treated with RLX-PL-MEL showed significant activation in the death receptor TNF-α, it increased by almost twofold (2.37±.08) compared to RLX (0.77 ± 0.02), and PL-MEL (1.8±.06) (Figure 10). This significant activation in TNF-α initiated programmed cell death following RLX-PL-MEL treatment.

### 3.11. NF-κB

**Figure 11.**
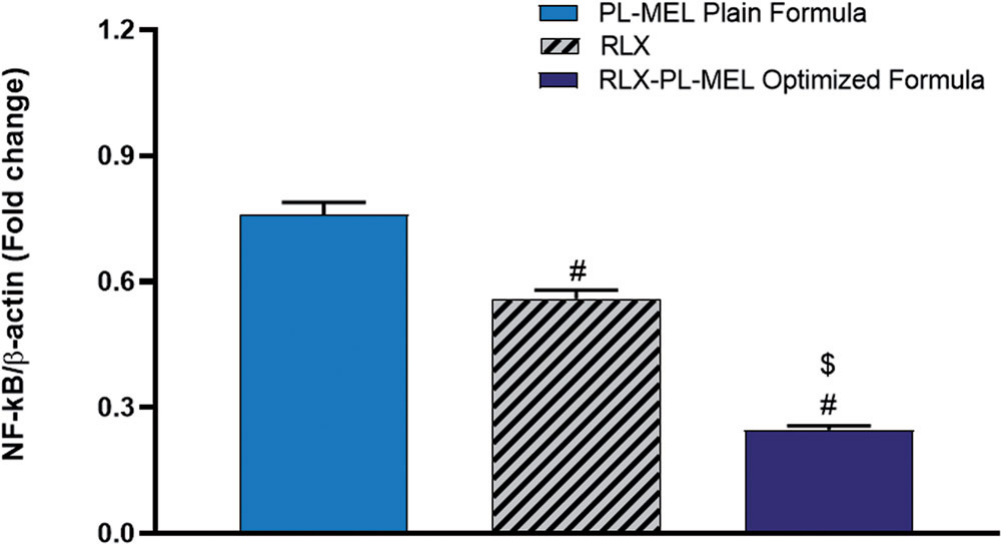
Effect of PL-MEL, RLX, or RLX-PL-MEL treatment on NF-κB activation. Data are expressed as the fold change of mean ± SD of three independent experiments where ^#,$^*p*<.0001. ^#^Significant from PL-MEL plain formula and ^$^significant from RLX treated. Samples were normalized to β actin.

Nuclear factor kappa-light-chain-enhancer of activated B cells (NF-κB) is a transcription factor and a key mediator of inflammation, which plays a role in tumor initiation and cancer cell proliferation (Aggarwal et al., 2019; Iqubal et al., 2020). Moreover, NF-κB upregulation has been frequently demonstrated in mice with PC (Iqubal et al., 2020). Hence, in this experiment, the transcription levels of NF-κB were examined using samples from PANC1 cells treated with PL-MEL, RLX, or RLX-PL-MEL formulation. Indeed, the optimized formula RLX-PL-MEL showed a significant downregulation in the NF-κB activity (0.24 ± 0.01) when compared to the drug alone RLX (0.55 ± 0.02) or the plain formula PL-MEL (0.75 ± 0.03) (*p*<.0001) (Figure 11). These results are in alignment with recently published data that demonstrated the inhibition of NF-κB actually enhances the antitumor activity of PC cells by downregulating anti-apoptotic protein Bcl-2 (Alhakamy et al., 2021). Once again, the downregulation of NF-κB in this experiment emphasizes the effectiveness of the optimized formula RLX-PL-MEL against the others in treating PANC1 cancer cells.

### 3.12. Mitochondrial membrane potential

**Figure 12.**
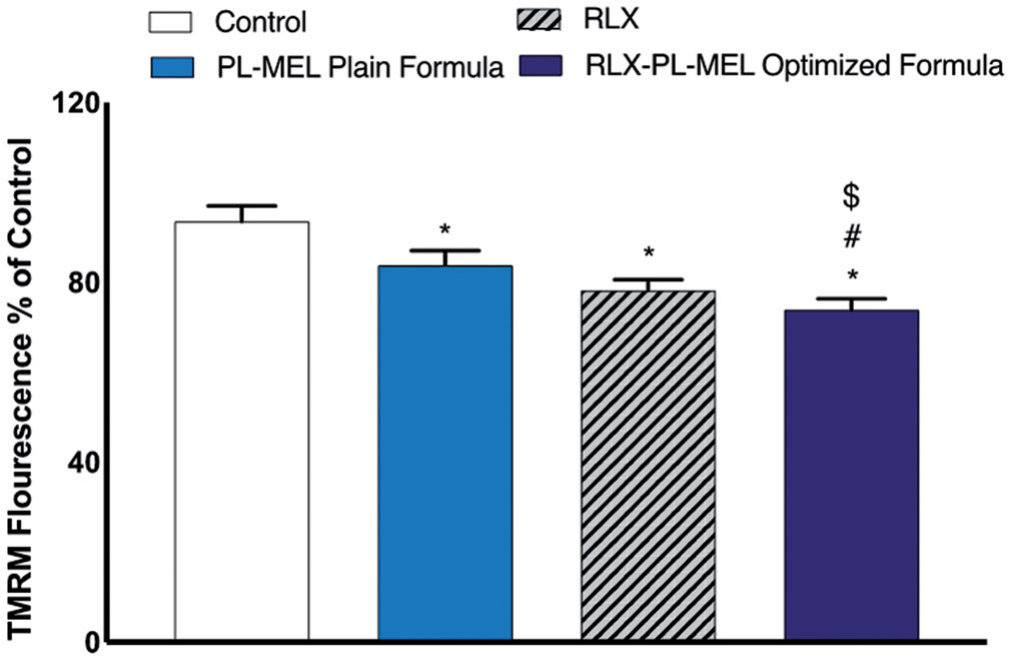
Changes in MMP of PANC1 treated with RLX, PL-MEL plain formula, and RLX-PL-MEL optimized formula. Data are expressed as percentage of mean ± SD of three independent experiments where *^,#,$^*p*<.0001. *Significant from control, ^#^significant from PL-MEL plain formula, and ^$^significant from RLX treated.

The loss of MMP was measured using flow cytometry to examine whether RLX, PL-MEL, and RLX-PL-MEL affect PANC1 cell apoptosis. Figure 12 shows that the MMP in the PANC1 cells treated with RLX-PL-MEL was significantly reduced compared to control, RLX, and PL-MEL. Although PANC1 cells treated with RLX, and PL-MEL demonstrated some MMP depletion, the effect of the optimized formula RLX-PL-MEL was more significant compared to these two, which suggest that RLX-PL-MEL potent apoptotic effect is mediated by the stimulation of the mitochondrial apoptosis pathway.

## 4. Discussion

PC prevalence and incidence have both increased exponentially in recent years. One of the most serious problems with PC is the presence of asymptotic manifestations, which frequently delays timely diagnosis, and until the diagnosis is made, tumor cells progress to the metastatic stage. Another significant concern with PC is the scarcity of well-defined pharmacotherapeutic drugs (Khalaf et al., 2021). Currently, only surgery is a standard regimen, and in other cases, a combination of surgery with chemotherapy and radiotherapy is used (McGuigan et al., 2018). The use of various chemotherapeutic agents, on the one hand, is associated with the serious adverse effect, whereas on the other hand, the deficit blood supply in the pancreatic region hinders the drug availability at the sight of the action (McGuigan et al., 2018). As a result, RLX-PL-MEL and a simple formula were created, optimized, and described in the current investigation. The formulation’s anticancer potential was evaluated in PANC1 cells using multiple apoptotic measures, MTT assay, expression of various genes, inflammatory markers, and cell cycle arrest analyses. A formula for the preparation of optimized RLX-PL-MEL was obtained from the results of response surface-based design experiment using the face-centered central composite design.

It is important to mention that PL not considered has cytotoxicity properties as mentioned in a previous work (Nassimi et al., 2009). It is well-known that carcinogenesis results from the imbalance between the cell cycle and death in response to carcinogens and alteration in the signaling pathways (Iqubal et al., 2020). RLX was approved by FDA for treatment of osteoporosis and the prevention of breast cancer. RLX acts by a tissue-specific manner through binding, with high affinity, to both ERα and ERβ and acting as either ER agonist, antagonist, or both (Pike et al., 1999; Pozios et al., 2021). RLX has shown efficacy against pancreatic adenocarcinoma growth through ERβ and the IL-6/gp130/STAT3 signaling pathway interference in PDAC cell line (Pozios et al., 2021). MEL has been widely investigated for utilization in cancer therapy, for reviews (Liu et al., 2016; Wang et al., 2022). MEL has demonstrated effectiveness in enhancing apoptosis through the enhancement of death receptor 3 expression and inhibition of NF-κB pathway in A549 and NCI-H460 cells and downregulating of ERK and Akt signaling pathway in leukemic U937 cells (Moon et al., 2006; Choi et al., 2014). The inhibition of JAK2/STAT3, and the Rac1-dependent pathway, and the activation of mitochondrial pathway was also reported (Liu et al., 2008; Jo et al., 2012; Kong et al., 2016). Nano formulation can preserve active pharmaceuticals agents in the bloodstream, enhance anticancer medication biodistribution, and target tumors (Vago et al., 2016). As a result, it is unavoidable that MEL transformed nanocarriers has attracted researchers’ interest. MEL functionalized lipidic nanovesicles could represent a promising approach for enhancement the toxicity of the payload (RLX). Endosomal escape of MEL conjugates with routinely used polymers for nucleic acid delivery has been widely demonstrated. Transfection tests on a number of cell lines revealed that MEL-PEI-luciferase DNA conjugates have up to 700-fold better transfection effectiveness than the control group (PEI-DNA conjugates). MEL was covalently bonded to poly(ethylenimine) (PEI) condensed DNA into tiny, discrete particles (100 nm in diameter). This conjugate’s transfection activity was significantly higher than PEI across a wide variety of cell lines and types (Moreno & Giralt, 2015; Liu et al., 2016).

Bcl-2 and Bcl-xL are one apoptotic protein and have been associated with cell survival of tumor cells via blocking the mechanism of programmed cell death. It was further reported that overexpressed Bcl2 was associated with proliferation and Myc-induced angiogenesis (Wong, 2011; Iqubal et al., 2020). On the other hand, Bax is a pro-apoptotic protein related to the induction of apoptosis, which coordinates with caspases leading to the death of tumor cells. Thus, an increased level of Bcl-2 is associated with anti-oncogenic activity, whereas an increased level of Bax is associated with pro-oncogenic activity. In the current study, when we checked the anticancer potential of RLX-raw, plain formula, and optimized RLX-PL-MEL showed reduced expression of Bcl-2 and increased expression of Bax in the PANC1 cells and hence confirmed the enhanced pro-apoptotic and anticancer potential of RLX-PL-MEL.

Additionally, it has been found that pro-apoptotic proteins such as Bax and pro-inflammatory cytokine TNF-α alter the MMP and alter the permeability transition pore (PTP) responsible for the release of cytochrome *c* from the outer mitochondrial membrane (Burke, 2017; Iqubal et al., 2019). Once cytochrome *c* is released from the mitochondrial pore, it associates with Apaf-1 and cascade for apoptosome formation, and caspases such as caspase 3 continues and results in apoptosis (Burke, 2017). Additionally, TNF-α binds with the TNF-α receptors and initiates the mechanism of extrinsic apoptosis via TNF receptor-associated death domain (TRADD) death-inducing signaling complex (DISC). In brief, binding of TNF-α with TNF-α receptor causes activation of procaspase-8 to caspase-8 that in turn convert procaspase-3 produced via Cyt c, Apaf-1, and apoptosome into caspase-3 and hence causes apoptosis (Josephs et al., 2018; Kretz et al., 2018). In the present study, exposure RLX-PL-MEL to the PANC1 cells showed reduced MMP, increased expression of TNF-α and caspase-3 and hence, signifies mitochondrial-mediated apoptosis. It is well established that the apoptotic potency of a drug candidate is validated by the success of an anticancer drug to arrest cycle. In the present study, using RLX-PL-MEL showed cell cycle arrest at the G2-M phase and confirmed the anticancer potential.

## 5. Conclusions

A promising nanovesicle formula of RLX loaded MEL functionalized PL nanovesicles was investigated for efficacy against PC cell line. This investigation yielded an optimized RLX-PL-MEL with minimum particle size, maximum zeta potential, and maximum entrapment efficiency %, which fulfilled the criteria of the objective for the selection of optimized nanovesicles via face-centered central composite design-based Design Expert Software (Stat-Ease Inc., Minneapolis, MN). In this case, obtained polynomial equations clearly showed the effects of various independent factors over responses. Afterward, the comparative therapeutic efficacy was established treating PANC1 cells with optimized RLX-PL-MEL, RLX-raw, and plain formula. Results illustrated enhanced anti-PC activity by intercepting cell cycle in G2-M phase, and superior apoptosis when compared with RLX or plain formula. Thus, the favorable results for RLX-PL-MEL, making it a novel promising treatment approach against PC.

## Author contributions

Conceptualization: UA, SMB, and HM; methodology: GA, NA, OA, BG, SR, and MF; software: WA; validation: GA, NA, and OA; formal analysis: UA, SMB, and HM; investigation: WA; resources: GA, NA, OA; data curation: BG, SR, MF; writing – original draft preparation: WA, UA, and SMB. Writing – review and editing: UA and SMB; visualization: NA; supervision: NA; project administration: WA; funding acquisition: UA and SMB. All authors have read and agreed to the published version of the manuscript. Please turn to the CRediT taxonomy for the term explanation. Authorship must be limited to those who have contributed substantially to the work reported.
